# Notes and News

**Published:** 1903-11-28

**Authors:** 


### Notes and News.

A dance is to be held in the Whitehall Rooms, Hôtel Métropole, under the patronage of the Princess Louise, the proceeds of which are to be devoted towards re flooring the wards of Charing Cross Hospital.

The annual Court of Governors of the Royal Waterloo Hospital for Children and Women, Waterloo Bridge Road, will take place at the Mansion House at 3 p.m. on Monday, January 18th, 1904. The president, the Right Hon. the Lord Mayor, will occupy the chair.

Sir Henry Burdett, K.C.B., will open a discussion on "London Hospitals and Medical Schools and their Sites" at a meeting of the Hospitals Association on Thursday, December 3rd, at 4.30 p.m., in the board-room of Charing Cross Hospital. Mr. Thomas Bryant (consulting surgeon of Guy's Hospital) will take the chair, and tickets may be had on application to Mr. Sydney Phillips at St. Thomas's Hospital.

The late Mr. F. W. Schlusser has bequeathed £1,000 each to the London Fever Hospital and the German Hospital. The late Miss E. Crowther has bequeathed £400 to the Leeds General Infirmary, £200 each to the Harrogate Royal Bath Hospital, the Ilkley Hospital, the Leeds Hospital for Women and Children, and the Cookridge Convalescent Home. The late Mr. R. Trimmer has bequeathed £3,000 to the Trimmer's Cottage Hospital, Farnham.

The authorities of the Royal Waterloo Hospital for Children and Women, Waterloo Bridge Road, hope to establish a military ward with the assistance of the various London corps. The name and regimental crest of each regiment is to be fixed at the head of each cot, and devoted to the sick children and wives of the rank and file. Six hundred and thirty shillings, or 30 guineas annually, will support a cot, or the capital sum of 1,000 guineas. Already one individual has generously promised to endow a cot on condition that five others will do the same.

Cambridge University is about to establish a diploma in tropical medicine.

Sir John Williams opened the new casualty department at the Cardiff Infirmary last week.

The King of Italy, through the Italian Ambassador, has sent the munificent donation of £1,000 to the Italian Hospital, Queen Square, as a special mark of his Majesty's approval of the hospital's work, and of his regret that time did not allow his Majesty to personally visit the institution.

Mr. Bayliss, who has just received £2,000 in the form of damages for libel from Mr. Stephen Coleridge, Hon. Sec. of the Anti-Vivisection Society, has stated that this sum "will be devoted, in some form not yet decided, to the furtherance of physiological research at University College."

Three cases of sleeping sickness have been brought from the Congo to Paris by Dr. Emile Brumpt, for the purpose of study. They have been placed in the Hôspital des Dames Françaises, at Auteuil, and the use of the hospital has been temporarily assigned to the Paris School of Tropical Medicine for the purpose of instruction.

An address was presented on behalf of the Committee of Management and staff of the Italian Hospital to their Majesties the King and Queen of Italy at the reception held at the Italian Embassy on Thursday morning. In addition to the address Mrs. Angiola Ortelli, the widow of the founder of the hospital, presented his Majesty the King with an album of views of the building.

Last week Sir William Broadbent made presentations to Sir Anderson Critchett and Mr. Malcolm Morris on their retirement from the medical staff of St. Mary's Hospital, after 20 years' active service. Sir Anderson Critchett received a pair of handsome candelabra, Mr. Malcolm Morris received 145 volumes of the "Annual Register," and jewellery was presented to Mrs. and Miss Morris.

The International Sanitary Conference at Paris has finished its labours. Turkey, Greece, and Bulgaria are not in accord with the other European countries in the matter of quarantine, which the rest of the Conference were in agreement should be almost abolished. The preventive measures recommended in regard to the introduction of disease included the extermination of rats. The establishment of an international sanitary bureau in Paris has been decided upon.

The memory of Sir John Blundell Maple, who died after a long illness on Tuesday morning, will be perpetuated in the hospital world by several generous benefactions. He was the donor of the new building of University College Hospital, which has already cost upwards of £150,000. In 1894 he and Lady Maple gave the city of St. Albans a small hospital, and last year he was the leading spirit in the foundation of the Victoria Memorial Hospital, which is now being built under his instructions at Mont Borou, Nice.

